# Relation between the Co-O bond lengths and the spin state of Co in layered Cobaltates: a high-pressure study

**DOI:** 10.1038/s41598-017-03950-z

**Published:** 2017-06-16

**Authors:** Yi-Ying Chin, Hong-Ji Lin, Zhiwei Hu, Chang-Yang Kuo, Daria Mikhailova, Jenn-Min Lee, Shu-Chih Haw, Shin-An Chen, Walter Schnelle, Hirofumi Ishii, Nozomu Hiraoka, Yen-Fa Liao, Ku-Ding Tsuei, Arata Tanaka, Liu Hao Tjeng, Chien-Te Chen, Jin-Ming Chen

**Affiliations:** 1 National Synchrotron Radiation Research Center, Hsinchu, 30076 Taiwan; 2 Max Planck Institute for Chemical Physics of Solids, Dresden, D-01187 Germany; 3 Karlsruhe Institute of Technology (KIT), Institute for Applied Materials (IAM), Eggenstein-Leopoldshafen, D-76344 Germany; 4 Institute for Complex Materials, IFW Dresden, Dresden, D-01069 Germany; 5 Department of Quantum Matter, ADSM, Hiroshima University, Higashi-Hiroshima, 739-8530 Japan

## Abstract

The pressure-response of the Co-O bond lengths and the spin state of Co ions in a hybrid 3d-5d solid-state oxide Sr_2_Co_0.5_Ir_0.5_O_4_ with a layered K_2_NiF_4_-type structure was studied by using hard X-ray absorption and emission spectroscopies. The Co-*K* and the Ir-*L*_*3*_ X-ray absorption spectra demonstrate that the Ir^5+^ and the Co^3+^ valence states at ambient conditions are not affected by pressure. The Co *Kβ* emission spectra, on the other hand, revealed a gradual spin state transition of Co^3+^ ions from a high-spin (S = 2) state at ambient pressure to a complete low-spin state (S = 0) at 40 GPa without crossing the intermediate spin state (S = 1). This can be well understood from our calculated phase diagram in which we consider the energies of the low spin, intermediate spin and high spin states of Co^3+^ ions as a function of the anisotropic distortion of the octahedral local coordination in the layered oxide. We infer that a short in-plane Co-O bond length (<1.90 Å) as well as a very large ratio of Co-O_apex_/Co-O_in-plane_ is needed to stabilize the IS Co^3+^, a situation which is rarely met in reality.

## Introduction

Layered perovskites A_2_BO_4_ with a K_2_NiF_4_-type structure have been intensively investigated owing to their unique properties, such as high-temperature superconductivity in cuprates, spin-triplet superconductivity in ruthenates, spin/charge stripes in nickelates and manganites^1^. Recently, Sr_2_IrO_4_ with low-spin (LS) Ir^4+^ has attracted much attention because of the insulating behavior resulting from the strong spin-orbit interaction^2, 3^, while Sr_2_CoO_4_ exhibits a metallic behavior because of its intermediate-spin (IS) Co^4+^ coming from both the negative charge-transfer energy and the tetragonal distortion^4–10^. In La_2-x_Sr_x_CoO_4_, the CoO_6_ octahedron has an elongated distortion, and thus the IS Co^3+^ state might be stabilized owing to the single occupation in the e_g_ levels. Therefore, the spin state of the Co^3+^ ions in La_2-x_Sr_x_CoO_4_ has been controversially discussed as a pure IS state or alternatively as a mixture of high spin (HS) Co^3+^ and low-spin (LS) Co^3+^ ^11–16^. There are also conflicting results in the pressure-driven spin crossover of Co^3+^ ion in the layered compound Sr_2_CoO_3_F with the K_2_NiF_4_-type structure^17^. First principle calculations predicted the HS state at ambient pressure and the IS state under high pressure^18^, while Co *Kβ* emission experiments suggested a complete HS-LS transition at 12 GPa without through an IS state^19^. Therefore, the presence of the IS Co^3+^ is still under fierce debate.

The hybrid Co/Ir solid-state oxide Sr_2_Ir_2-x_Co_x_O_4_ system might show unusual electronic and magnetic structures considering the presence of strong intra-atomic multiplet interactions for the localized Co 3d electrons and a large spin-orbit coupling for the delocalized Ir 5d electrons. As indicated by a previous study, the substitution of Ti, Fe, and Co for Ir in Sr_2_IrO_4_ induces a reduction of the magnetic susceptibility as well as an enhancement of the effective paramagnetic moment for samples with Co and Fe together with a suppression of the weak ferromagnetic ordering^20^. On the other hand, substituting Mn for Ir results in the reordering and flipping of the spins as well as a decrease of the magnetic ordering temperature^21^. Co in Sr_2_Ir_1-x_Co_x_O_4_ is proposed to be in 4 + valence state for Co concentrations up to 30%, while the effective magnetic moment (4.69 μ_B_) falls in between what is expected for IS Co^4+^ (3.87 μ_B_) and high spin (HS) Co^4+^ (5.92 μ_B_)^20^. However, a later theoretical study proposed the presence of a charge-spin-orbital state in Fe- or Co-doped Sr_2_IrO_4_ with HS Fe^3+^ and HS Co^3+^ instead of IS Fe^4+^ and IS Co^4+^ ^22^. The spin state degree of freedom of Co results from subtle balance between crystal field splitting and Hund’s rule exchange energy. Pure Low-spin (LS) Co^3+^ is well known, such as LiCoO_2_, NaCoO_2_, and EuCoO_3_, while pure high-spin (HS) Co^3+^ exists only in systems with the relatively weak crystal field like YBa_2_Co_4_O_7_ with CoO_4_ tetrahedrons^23^ and Sr_2_CoO_3_Cl with CoO_5_ pyramids^24^. The HS Co^3+^ with CoO_6_ symmetry in cobalt oxides was only found in the system with a mixture of HS and LS like LaCoO_3_^25^ or in the system with oxygen deficiency such as GdBaCo_2_O_5.5_^26^. Considering that Sr_2_IrO_4_ has relatively large lattice parameters (Ir-O_in-plane_ = 1.9832 Å)^27^, it is expected that the Co^3+^ ions doped in Sr_2_IrO_4_ would be in a pure HS state owing to the weak crystal field. However, pressure dependence of crystal-structure study on Sr_2_Co_0.5_Ir_0.5_O_4_ has shown a sharp increase of the *c/a* ratio with pressures up to 10 GPa^28^. This increase in the tetragonal distortion should favor the IS Co^3+^ state. Furthermore, Sr_2_Co_0.5_Ir_0.5_O_4_ exhibits a negative Weiss constant, indicating a dominant antiferromagnetic interaction in this system^28^, which might be related to the spin state of Co. In this work, we have investigated the relation between the Co-O bond lengths and the spin states of Co^3+^ ions in Sr_2_Co_0.5_Ir_0.5_O_4_ under external pressures. We have drawn a phase diagram of the spin state of a Co^3+^ ion as a function of the anisotropic Co-O bond lengths.

## Results

### Co-*L*_2,3_ X-ray absorption

**Figure 1:**
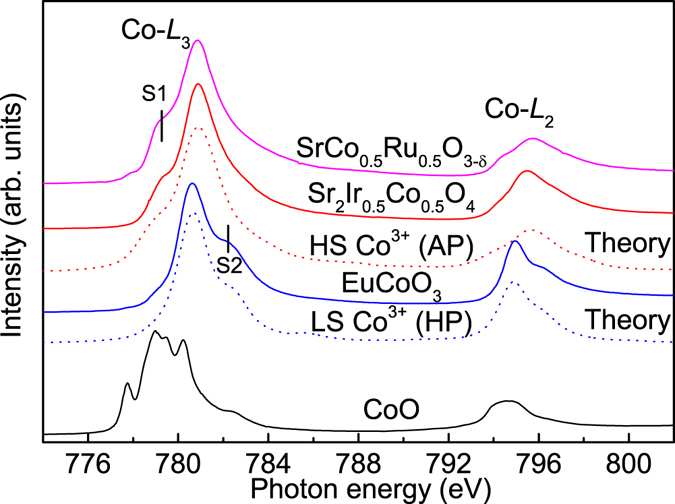
Co-*L*_2,3_ absorption spectra of Sr_2_Co_0.5_Ir_0.5_O_4_ together with EuCoO_3_ as a LS-Co^3+^ ref. 24, SrCo_0.5_Ru_0.5_O_3-δ_ as a HS-Co^3+^ ref. 29, and CoO as a HS-Co^2+^ reference. The theoretical spectra of HS Co^3+^ below Sr_2_Co_0.5_Ir_0.5_O_4_ and LS Co^3+^ below EuCoO_3_ are also included for comparison.

The Co-*L*_2,3_ XAS spectrum of Sr_2_Co_0.5_Ir_0.5_O_4_ is presented in Fig. 1 together with those of EuCoO_3_ as a LS-Co^3+^ reference, SrCo_0.5_Ru_0.5_O_3-δ_ as a HS-Co^3+^ reference, and CoO as a high-spin (HS) Co^2+^ ^24, 29^. One can see that the center of gravity of the *L*_3_ white line of Sr_2_Co_0.5_Ir_0.5_O_4_ (red line) is at a higher photon energy as compared to that of CoO, while it is similar to that of EuCoO_3_ and SrCo_0.5_Ru_0.5_O_3-δ_. This establishes that the Co in Sr_2_Co_0.5_Ir_0.5_O_4_ is trivalent, different from the parent compound Sr_2_CoO_4_ with Co^4+^. Moreover, the line shape of the Sr_2_Co_0.5_Ir_0.5_O_4_ spectrum is very different from that of EuCoO_3_, implying a different local electronic structure. As shown in previous studies, the presence of the low-energy shoulder S1 at the Co^3+^ *L*_3_ edge is characteristic for the high-spin state, while the high-energy shoulder S2 is indicative for the low-spin state^24, 29^. The similarity between Sr_2_Co_0.5_Ir_0.5_O_4_ and SrCo_0.5_Ru_0.5_O_3-δ_ also shows the same spin state, namely HS. To further confirm HS Co^3+^ in Sr_2_Co_0.5_Ir_0.5_O_4_, we performed the configuration-interaction cluster calculations including the full atomic multiplet, and the crystal field interactions, as well as the hybridization between the Co and oxygen ions according to Harrison’s presscription^30, 31^. The parameter values are listed in ref. 32. The theoretical HS Co^3+^ spectrum was plotted below Sr_2_Co_0.5_Ir_0.5_O_4_. One can observe that the HS-Co^3+^ scenario nicely reproduces all features of the experimental spectrum, further demonstrating the HS Co^3+^ ground state in this system. We would like to note that the 3 + valence of the Co is fully consistent with the finding of the 5 + valence of the Ir ion as demonstrated in the previous study by the Ir-*L*_3_ XAS spectrum^28^.

### Co-*K* X-ray absorption under pressure

**Figure 2:**
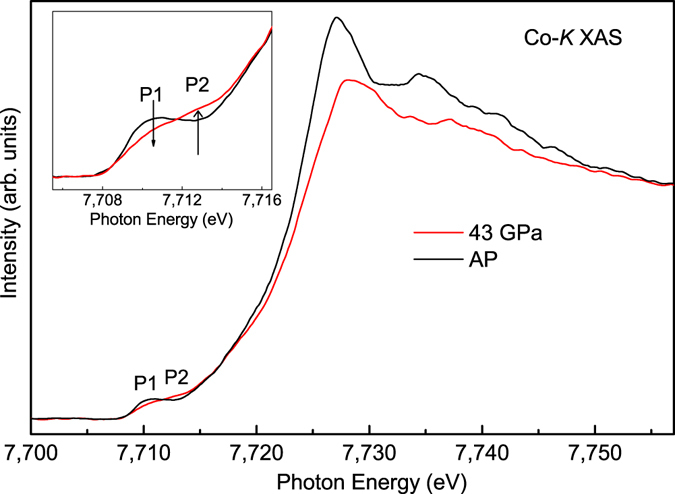
The Co-*K* PFY XAS spectra of Sr_2_Co_0.5_Ir_0.5_O_4_ at ambient pressure and 43 GPa.

We now investigate the Co spin state as a function of pressure using hard X-rays. The spin state can be determined also by the Co-*K* XAS spectra, since different spin states possess distinct electronic structures. The Co-*K* XAS spectra at ambient pressure and at 43 GPa are shown in Fig. 2. The XAS spectra contain two broad features in the pre-edge region around 7,710 eV, and one intense absorption peak around 7,725 eV. The main peak can be attributed to the dipole transition from the Co 1 s core level to the Co 4p unoccupied states, while the pre-edge structures can be assigned to transitions from the Co 1 s to the Co 3d t_2g_ and e_g_ levels owing to the hybridization between Co 3d and 4p states^33^. As shown in the inset of Fig. 2, one observes a spectral weight transfer with pressure: the low-energy feature P1 loses its spectral intensity, while the feature P2 gains its spectral intensity. As indicated by the charge-transfer multiplet calculation in an earlier study^34^, the LS state has only one single peak in the pre-edge range, while both the IS and HS states possess two features because of the accessible t_2g_ levels in the higher spin states. Since the IS and HS states only have relatively small line shape differences, the strong spectral change implies the increase of the LS content with pressure^34^. Moreover, the raising edge is also shifted to higher photon energies with pressure. This shift is consistent with the spin state transition from the HS Co^3+^ to LS Co^3+^, since the latter has a larger band gap. All this is consistent with the findings of the temperature-dependence Co-*K* XAS studies on LaCoO_3_ and (Pr_0.7_Sm_0.3_)_0.7_Ca_0.3_CoO_3_^34, 35^, in which the Co-*K* absorption edge of the low spin Co^3+^ at the low temperature is at higher photon energies compared to that of the higher spin Co^3+^.

### Co-*K* X-ray emission under pressure

**Figure 3:**
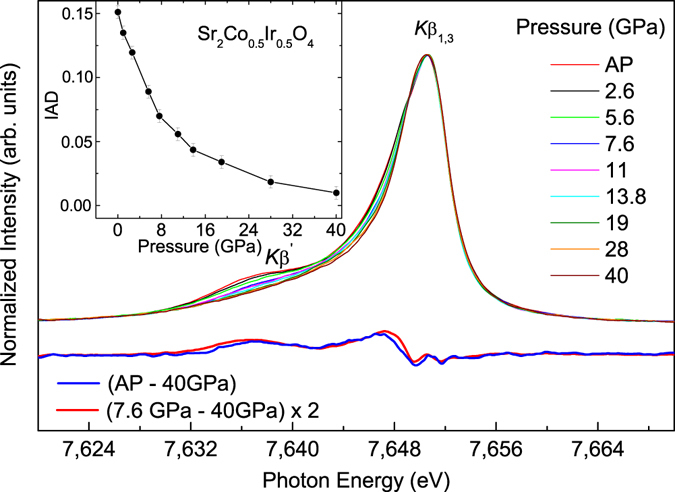
Co *Kβ* X-ray emission spectra (XES) of Sr_2_Co_0.5_Ir_0.5_O_4_ and difference spectra of Co *Kβ* emissions between ambient pressure (AP) and 40 GPa (blue line) and between 7.6 and 40 GPa (red line). Inset: Integrated absolute difference (IAD) as a function of pressure for Sr_2_Co_0.5_Ir_0.5_O_4_.

**Figure 4:**
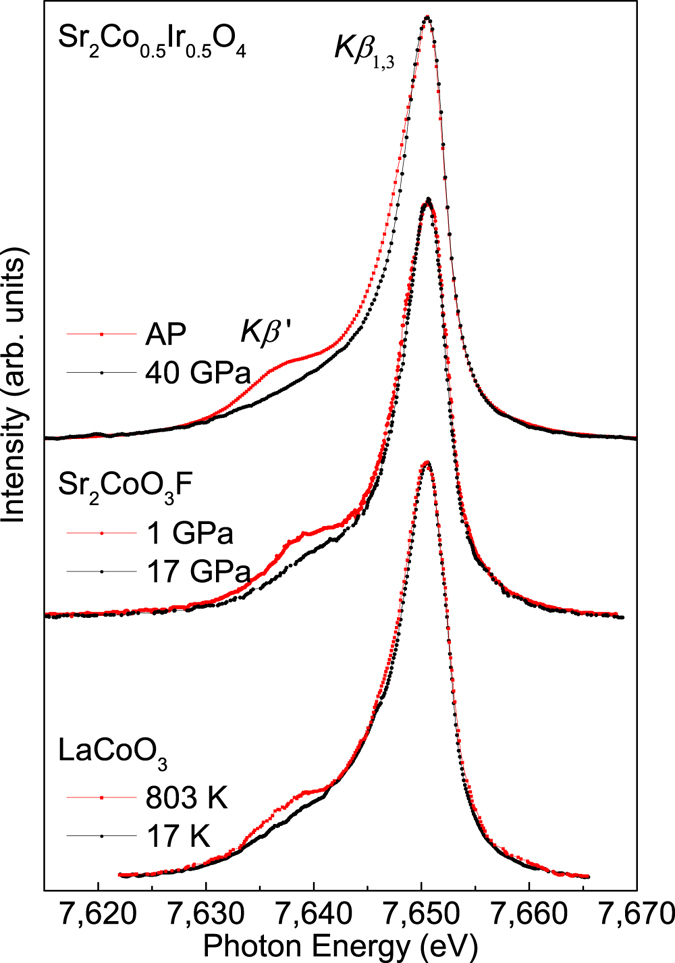
Co *Kβ* X-ray emission spectra (XES) of Sr_2_Co_0.5_Ir_0.5_O_4_ at ambient pressure (red line) and 40 GPa (blue line) together with those of Sr_2_CoO_3_F^19^ at 1 GPa (HS) and 17 GPa (LS) and those of LaCoO_3_^34^ at 17 K (LS) and 803 K (mainly HS).

To identify the pressure-induced spin state transition of HS-Co^3+^, we have collected the Co-*Kβ* emission spectra of Sr_2_Co_0.5_Ir_0.5_O_4_ in the pressure range between ambient pressure and 40 GPa as shown in Fig. 3. The ambient-pressure Co *Kβ* emission spectrum represents a main peak located at ~7,650 eV corresponding to the *Kβ*_1,3_ line, and a pronounced satellite peak at ~7,637 eV corresponding to the *K*β′ line. This line shape is typical for the HS-Co^3+^ state, as obtained in the compounds with HS-Co^3+^ like SrCo_0.5_Ru_0.5_O_3-δ_^29^ or LaCoO_3_ at high temperature^34^. The intensity ratio of the low-energy *Kβ*′ line to the main emission *Kβ*_1,3_ line is proportional to the number of the unpaired electrons in the incomplete 3d shell^36^ and can be used for an indication of spin states in the material^29, 33–35^. With increasing pressure, the intensity of the low-energy *Kβ*′ line decreases and almost disappears at 40 GPa (Fig. 3). Figure 4 presents the Co *Kβ* XES data of Sr_2_Co_0.5_Ir_0.5_O_4_ at AP and 40 GPa together with those of Sr_2_CoO_3_F at 1 GPa (HS) and 17 GPa (LS) as well as those of LaCoO_3_ at 17 K (LS) and 803 K (mainly HS)^19, 34^. To compare the intensity ratio of the *Kβ*_1,3_ line and the *Kβ*′ line, those data are aligned and normalized to the *Kβ*_1,3_ peak. As shown in Fig. 4, the reduction of the *Kβ*′ spectral weight in Sr_2_Co_0.5_Ir_0.5_O_4_ is the same as that of Sr_2_CoO_3_F^19^ indicating the complete HS-LS state transition in Sr_2_Co_0.5_Ir_0.5_O_4_ up to 40 GPa. But the decrease of the *K*β′ spectral weight is much larger than that of LaCoO_3_^34^ from 803 K to 17 K, since the spin state transition in the latter is not complete in this temperature range. Furthermore, the inset of Fig. 3 presents integrated absolute difference (IAD) as a function of pressure^33–35^, and the total IAD changes by about 0.14 from ambient pressure to 40 GPa. This value is similar to that of SrCo_0.5_Ru_0.5_O_3-δ_^29^ and consistent with what is expected for a complete HS (S = 2) to LS (S = 0) transition^37^.

At 7.6 GPa, the IAD value of ∼0.07 corresponds to the change in the spin state Δ*S* = 1, comparing with the value at ambient pressure. Two possible scenarios may satisfy the averaged spin state with *S* = 1: either the existence of intermediate spin state of Co^3+^ (IS-Co^3+^, S = 1) or a coexistence of equal amounts of HS-Co^3+^ (S = 2) and LS-Co^3+^ (S = 0). The presence of IS-Co^3+^ in perovskite-like oxides is a matter of long-time discussions, especially for LaCoO_3_^34, 38, 39^ and other rare-earth metal cobaltates^40^. In the case of layered perovskites, the reported results about spin-state crossover of the Co^3+^ ions are also controversial: for example, upon replacement of La^3+^ by the larger Sr^2+^ in La_2-x_Sr_x_CoO_4_ a drastic change of magnetic and electronic properties was ascribed to a spin-state transition of Co^3+^ from a high-spin to an intermediate-spin^41^. On the other hand, spin state transition from the LS-Co^3+^ to HS-Co^3+^ upon the increase of temperature was reported for single crystals of La_2−x_Sr_x_CoO_4_, based also on susceptibility data analysis^14^.

In order to distinguish between two scenarios of possible Co^3+^ spin state in the layered Sr_2_Co_0.5_Ir_0.5_O_4_ at 7.6 GPa with the total spin state S = 1, namely a mixture of HS-Co^3+^ and LS-Co^3+^ and pure IS-Co^3+^, we drew the difference spectra of Co-*Kβ* emissions obtained between ambient pressure (AP) and 40 GPa (red line) as well as between 7.6 GPa and 40 GPa (blue line) shown in Fig. 3 (below the X-ray emission spectra). The red line corresponds to the change in the spin number Δ*S* = 2, while the blue line describes the change in the spin number Δ*S* = 1. These two difference spectra are almost identical apart from the scale factor of 2, used for the blue line, what is consequent with the scenario “1:1 mixture of HS-Co^3+^ and LS-Co^3+^ at 7.6 GPa”. Thus, the difference of the spectra does not show any sign for new features which would be expected for the presence of an intermediate spin state of Co^3+^. Therefore, a continuous spin state transition from HS-Co^3+^ to LS-Co^3+^ under pressures in Sr_2_Co_0.5_Ir_0.5_O_4_ can be verified. Note that in contrast to the nearly monotonous change of the IAD of SrCo_0.5_Ru_0.5_O_3_ with the pressure^29^, in the case of Sr_2_Co_0.5_Ir_0.5_O_4_ the IAD decreases fast up to 10–12 GPa following by slower decreasing at higher pressures. It might be related to the anisotropy compression of Sr_2_Co_0.5_Ir_0.5_O_4_ observed in the previous study^28^.

### Ir-*L*_*3*_ X-ray absorption under pressure

**Figure 5:**
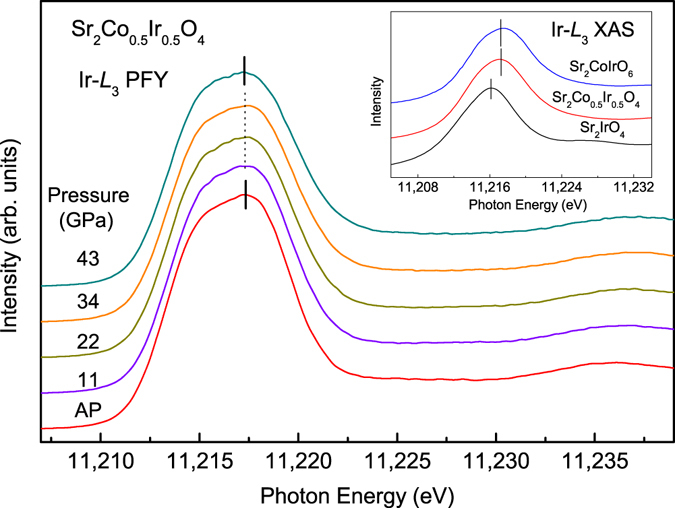
Pressure-dependence of the Ir-*L*_3_ PFY spectra of Sr_2_Co_0.5_Ir_0.5_O_4_. Inset shows the Ir-*L*_3_ XAS spectra of Sr_2_Co_0.5_Ir_0.5_O_4_, Sr_2_IrO_4_ as an Ir^4+^ reference and of Sr_2_CoIrO_6_^42^ as an Ir^5+^ reference for comparison.

At this point we also would like to know whether the reduction of Co spin moment under pressure results from a change of the Co configuration from HS Co^3+^ (S = 2) to LS Co^4+^ (S = 1/2) accompanying with a change of the Ir valence state from 5 + to 4 + . For this purpose, we measured the partial fluorescence spectra at the Ir–*L*_3_ edge of Sr_2_Co_0.5_Ir_0.5_O_4_ under pressures up to 43 GPa. As shown in Fig. 5, from bottom to top, there is no energy shift of the Ir-*L*_3_ PFY XAS spectra with the external pressures from AP to 43 GPa, indicating that the Ir valence remains 5 + , since a reduction of Ir valence state would lead to an energy shift to lower photon energies. As shown in inset of Fig. 5, the Ir-*L*_3_ XAS spectrum of Sr_2_Co_0.5_Ir_0.5_O_4_ measured in a transmission mode at ambient pressure is at higher photon energies compared with that of Sr_2_IrO_4_ with Ir^4+^, but locates at nearly the same photon energy as that of Sr_2_CoIrO_6_ with Ir^5+^ ^42^. Thus, we reaffirm that the decrease of the cobalt moment under pressure is solely due to a gradual spin state transition of Co^3+^ ions without any change in the valence state of the Co ions and also reaffirm the Ir^5+^ valence state, fulfilling the charge balance requirement for Co^3+^/Ir^5+^ valence states in the studied Sr_2_Co_0.5_Ir_0.5_O_4_ sample.

## Discussion

**Figure 6:**
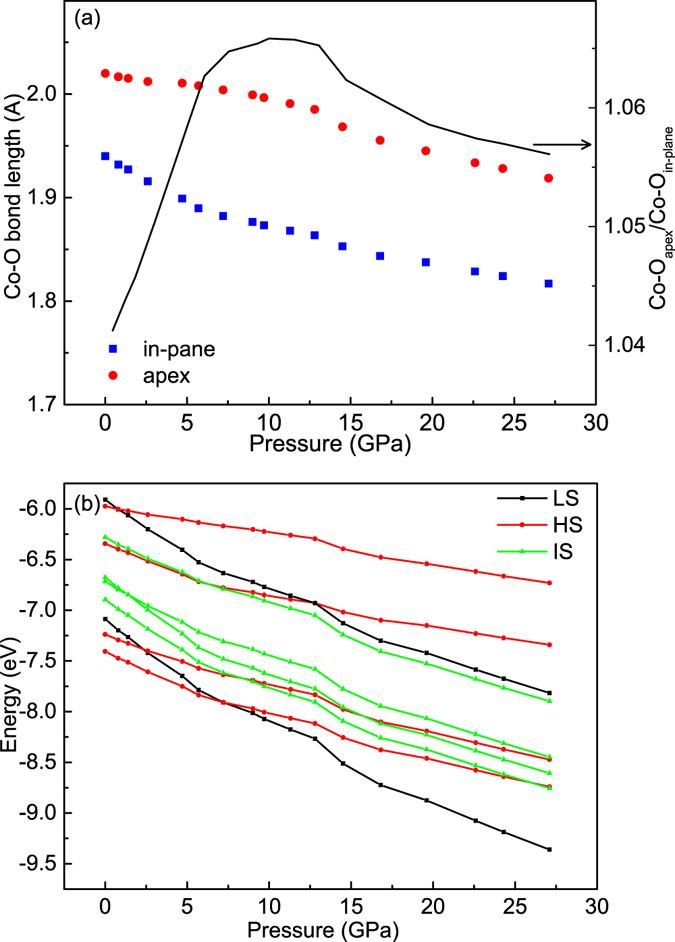
(**a**) Co-O_apex_ bond length (red circles) and Co-O_in-plane_ bond length (blue squares) as well as the ratio Co-O_apex_/Co-O_in-plane_ (black line) as a function of the external pressure derived from ref. 28. Note that the reduction of the in-plane Co-O bond distance might be overestimated due to the possible rotation of the CoO_6_ octahedron. (**b**) The energy diagram of three spin states as a function of the pressure using Co-O bond lengths in (**a**).

Using the element selective Co-*K* EXAFS (extended X-ray absorption fine structure) we can determine Co-O distance at ambient pressure^28^. If we assumed that the pressure-induced variation of the Co-O bond lengths would be proportional to the variation of the lattice parameters, then we estimated Co-O bond lengths as a function of pressure from the lattice parameters obtained in the previous high pressure study^28^, as presented in Fig. 6(a). Please note that under external pressures, a CoO_6_ octahedron might rotate in the basal plane, as observed in the study on Sr_2_RuO_4_ and Sr_2_IrO_4_^27^. Therefore, the reduction of the in-plane Co-O bond distances might be overestimated. However, our theoretical predication of the total energies of HS, LS and IS states as a function of in-plane and out-plane Co-O distances response to external pressure in general is still valid. One can see that the in-plane Co-O distance (Co-O_in-plane_ blue squares) reduces faster than that for the apex (Co-O_apex_ red circles) up to 10 GPa, namely Co-O_apex_/Co-O_in-plane_ (black line) increases with high pressure^26^. One would expect that the IS ground state of Co^3+^ ion with one electron in e_g_ orbital could be stabilized under high pressure as the tetragonal distortion increases with high pressure. It is puzzled, however, our above Co *Kβ* X-ray emission spectra indicate a pressure-induced spin state transition from the HS state to the LS state without crossing the IS state.

To understand above experimental observation on the spin state transition, we have calculated the total energies of the LS, IS, and HS states as a function of pressure by taking the estimated Co-O_apex_ bond length and Co-O_in-plane_ bond length into account using the configuration-interaction cluster calculation. The hybridization part is obtained according to the Harrison’s rules and the ionic crystal field is calculated as the Madelung potential. The factor of the Madelung potential can be determined because the HS and LS states are degenerated at 7.6 GPa, as observed in the Co *Kβ* X-ray emission spectra. The results are presented in Fig. 6(b). One can see in Fig. 6(b) that at ambient pressure, the ground state is the HS state consistent with the experimental Co-*L*_2,3_ XAS and Co *Kβ* X-ray emission results. Under external pressures up to 7.6 GPa, the LS and IS states gain more energies than the HS state due to the increase of 10 Dq because of a reduction of the Co-O bond lengths and to the enhancement of the e_g_ splitting (∆e_g_) from an increase of Co-O_apex_/Co-O_in-plane_, respectively. However, the energy gain of the LS Co^3+^ state (−24 Dq) overwhelms that of the IS Co^3+^ state (−14Dq-0.5∆e_g_). Therefore, as presented in Fig. 6(b), the LS state becomes the ground state when Co-O_apex_/Co-O_in-plane_ is larger than 1.065 at the pressure about 7.6 GPa. When the external pressure is larger than 9.7 GPa, the LS state becomes even more stable against the HS and the IS owing to the further increase in 10Dq and also a reduction of ∆e_g_ as Co-O_apex_/Co-O_in-plane_ decreases. As shown in Fig. 6(b), the IS state will never be the ground state under the pressure performed for the layered Sr_2_Co_0.5_Ir_0.5_O_4_, and thus one might wonder what is the condition to stabilize the IS state as a ground state for Co^3+^.

**Figure 7:**
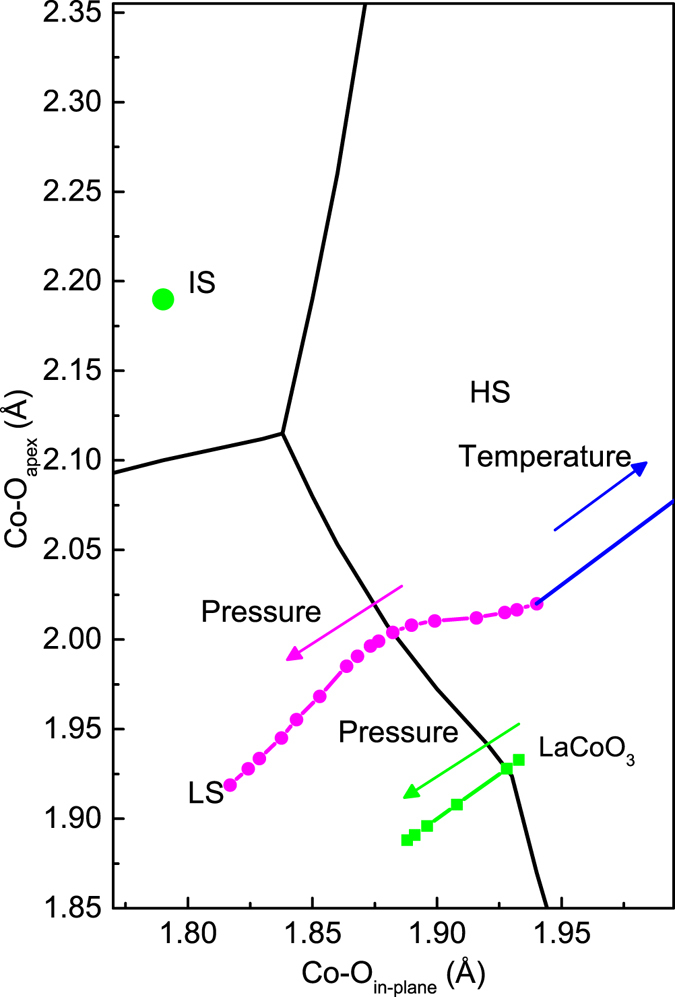
The phase diagram of the ground state of Co^3+^ as a function of Co-O bond lengths. The magenta circles are the Co-O bond lengths of Sr_2_Co_0.5_Ir_0.5_O_4_ estimated from ref. 28 and the blue line refers to an increase of its Co-O distance with temperature by keeping the ratio of Co-O_apex_/Co-O_in-plane_ at room temperature. The green circle is those of one of two Co^3+^ sites in the O-phase in TlSr_2_CoO_5_^43^. The green line represents the Co-O distances of LaCoO_3_^39^ with pressure.

In order to scrutinize the stable conditions for the IS Co^3+^ state in the layered structure, we have calculated the phase diagram of the ground state as a function of Co-O_apex_ and Co-O_in-plane_. The phase diagram shown in Fig. 7 indicates that the strong elongated tetragonal distortion indeed could stabilize the IS state if the in-plane Co-O distance (Co-O_in-plane_) is rather short and ratio of Co-O_apex_/Co-O_in-plane_ is quite large. In other words, the short in-plane Co-O bond length as well as the strong tetragonal distortion favors IS. However, if the Co-O_in-plane_ is larger than 1.90 Å, the IS state will be hardly stabilized. Therefore, IS cannot be stabilized by heating the sample as illustrated with the blue line where the Co-O distance increases with temperature by keeping the ratio of Co-O_apex_/Co-O_in-plane_ at room temperature in Fig. 7. On the other hand, the presence of the IS ground state might be possible in TlSr_2_CoO_5_, where one of two Co^3+^ sites at low temperatures (O-phase) has a small value of the Co-O_in-plane_ = 1.79 Å and Co-O_apex_ = 2.19 Å, presented as a green circle in Fig. 7 ^43, 44^. Besides, the Co-O bond lengths (magenta circles) in Sr_2_Co_0.5_Ir_0.5_O_4_ under external pressures are plotted in this phase diagram. One can see that the ground state of Co^3+^ ion in Sr_2_Co_0.5_Ir_0.5_O_4_ has a stable HS state and is transformed to the LS state with the external pressures without crossing the IS state (magenta circles). On the other hand, the Co-O bond distance of LaCoO_3_ is reduced from 1.9329 Å to 1.888 Å crossing a mixed HS/LS state to a pure LS state with pressure^39^. The mixed spin state of LaCoO_3_ is due to the much shorter Co-O bond distance, close to the boundary of the HS and LS, as compared with that of Sr_2_Co_0.5_Ir_0.5_O_4_ (Co-O_in-plane_ = 1.967 Å and Co-O_apex_ = 2.020 Å)^28^.

## Conclusion

We have studied the valence state and spin state transition of Co ion under external pressures in a hybrid 3d-5d transition metals solid-state oxide Sr_2_Co_0.5_Ir_0.5_O_4_ using hard X-ray absorption and Co-*Kβ* emission spectroscopies. The high spin state of Co^3+^ ions found at ambient pressure exhibits a complete spin state transition to the low-spin state up to 40 GPa without crossing the intermediate-spin state, while the valence state of Ir^5+^ ions remains unchanged. At external pressures below 9.7 GPa, the fast increase of the ratio of Co-O_apex_/Co-O _in-plane_ does not stabilize the IS state but the LS state instead owing to a rapid increase of 10Dq overwhelming the Jahn-Teller distortion of the e_g_ orbitals. Above 9.7 GPa, the LS state becomes even more stable due to the decrease of the ratio of Co-O_apex_/Co-O _in-plane_. To determine the condition for stabilizing a possible intermediate-spin ground state in such a layered oxide, we have compared the energies of the three different spin states of Co^3+^ ions as a function of bond lengths. These results have been plotted in a phase diagram and a stable IS state can only be found when the in-plane Co bond length is substantially shorter than the Co-O_apex_ bond length.

## Methods

### Sample synthesis

The layered polycrystalline Sr_2_Co_0.5_Ir_0.5_O_4_ was synthesized from solid state reaction as described previously^28^. The purity and unit cell parameters were determined by X-ray powder diffraction (XPD). Sr_2_Co_0.5_Ir_0.5_O_4_ is more insulating than Sr_2_IrO_4_^45^, as indicated by the resistivity data in Fig. S1 in the Supplementary Information.

### X-Ray spectroscopy

The Co-*L*_2,3_ X-ray absorption spectroscopy (XAS) measurements were recorded at the BL11A beam line of the National Synchrotron Radiation Research Center (NSRRC) in Taiwan. Clean sample surfaces were obtained by cleaving pelletized samples *in situ* in an ultra-high vacuum chamber with a pressure of 10^−10^ mbar range. The Co-*L*_2,3_ spectra were collected at room temperature using total electron yield mode (TEY) with an energy resolution of about 0.3 eV. The high-pressure Co-*K* and Ir-*L*_3_ partial-fluorescence-yield (PFY) XAS spectra and Co *Kβ* X-ray emission spectra were obtained at the Taiwan inelastic X-ray scattering BL12XU beamline at SPring-8 in Japan. A Mao-Bell diamond anvil cell with a Be gasket was used for the high-pressure experiment. Silicone oil served as a medium to transmit pressure. The applied pressure in the diamond anvil cell was measured through the Raman line shift of ruby luminescence before and after each spectral collection. The Co *Kβ* X-ray emission spectra were collected at 90° from the incident X-ray and analyzed with a spectrometer (Johann type) equipped with a spherically bent Ge(444) crystal and Si(553) (radius 1 m), respectively, arranged on a horizontal plane in a Rowland-circle geometry.

## Electronic supplementary material

Supplementary information.

## References

[1] Imada M, Fujimori A, Tokura Y (1998). Metal-insulator transitions. Rev. Mod. Phys..

[2] Kim BJ (2008). Novel Jeff=1/2 Mott State Induced by Relativistic Spin-Orbit Coupling in Sr_2_IrO_4_. Phys. Rev. Lett..

[3] Kim BJ (2009). Phase-Sensitive Observation of a Spin-Orbital Mott State in Sr_2_IrO_4_. Science.

[4] Matsuno J (2004). Metallic Ferromagnet with Square-Lattice CoO_2_ Sheets. Phys. Rev. Lett..

[5] Wang XL, Takayama-Muromachi E (2005). Magnetic and transport properties of the layered perovskite system Sr_2−y_Y_y_CoO_4_ (0≤y≤1). Phys. Rev. B.

[6] Matsuno J (2005). Novel metallic ferromagnet Sr_2_CoO_4_. Thin Solid Films.

[7] Matsuno J, Okimoto Y, Kawasaki M, Tokura Y (2005). Variation of the Electronic Structure in Systematically Synthesized Sr_2_MO_4_ (M = Ti, V, Cr, Mn, and Co). Phys. Rev. Lett..

[8] Lee K-W, Pickett WE (2006). Correlation effects in the high formal oxidation-state compound Sr_2_CoO_4_. Phys. Rev. B.

[9] Pandey SK (2010). Correlation induced half-metallicity in a ferromagnetic single-layered compound: Sr_2_CoO_4_. Phys. Rev. B.

[10] Wu H (2012). Metal-insulator transition in Sr_2−y_La_x_CoO_4_ driven by spin-state transition. Phys. Rev. B.

[11] Shimada Y, Miyasaka S, Kumai R, Tokura Y (2006). Semiconducting ferromagnetic states in La_1−x_Sr_1+x_CoO_4_. Phys. Rev. B.

[12] Chichev AV (2006). Structural, magnetic, and transport properties of the single-layered perovskites La_2−x_Sr_x_CoO_4_ (x = 1.0–1.4). Phys. Rev. B.

[13] Wu H (2010). High-spin and low-spin mixed state in LaSrCoO_4_: An *ab initio* study. Phys. Rev. B.

[14] Hollmann N (2011). Evidence for a temperature-induced spin-state transition of Co^3+^ in La_2−x_Sr_x_CoO_4_. Phys. Rev. B.

[15] Merz M (2011). Spin and orbital states in single-layered La_2−y_Ca_x_CoO_4_ studied by doping- and temperature-dependent near-edge x-ray absorption fine structure. Phys. Rev. B.

[16] Moritomo Y, Higashi K, Matsuda K, Nakamura A (1997). Spin-state transition in layered perovskite cobalt oxides: La_2−y_Sr_x_CoO_4_ (0.4≤x≤1.0). Phys. Rev. B.

[17] Tsujimoto Y (2012). Crystal Structural, Magnetic, and Transport Properties of Layered Cobalt Oxyfluorides, Sr_2_CoO_3+x_F_1–x_ (0≤x≤0.15). Inorg. Chem..

[18] Ou X, Fan F, Li Z, Wang H, Wu H (2016). Spin-state transition induced half metallicity in a cobaltate from first principles. Appl. Phys. Lett..

[19] Tsujimoto, Y. *et al*. Crystal Pressure-Driven Spin Crossover Involving Polyhedral Transformation in Layered Perovskite Cobalt Oxyfluoride. *Sci. Rep*. **6**, Article number: 36253 (2016*)*. 10.1038/srep36253 PMC5090247 27805031

[20] Gatimu AJ, Berthelot R, Muir S, Sleight AW, Subramanian MA (2012). Synthesis and characterization of Sr_2_Ir_1–x_M_x_O_4_ (M = Ti, Fe, Co) solid solutions. J. Solid State Chem..

[21] Calder S (2012). Magnetic structural change of Sr_2_IrO_4_ upon Mn doping. Phys. Rev. B.

[22] Ou X, Wu H (2014). Coupled charge-spin-orbital state in Fe- or Co-doped Sr_2_IrO_4_. Phys. Rev. B.

[23] Hollmann N (2009). Electronic and magnetic properties of the kagome systems YBaCo_4_O_7_ and YBaCo_3_MO_7_ (M = Al, Fe). Phys. Rev. B.

[24] Hu Z (2004). Different Look at the Spin State of Co^3+^ Ions in a CoO_5_ Pyramidal Coordination. Phys. Rev. Lett..

[25] Haverkort MW (2006). Spin State Transition in LaCoO_3_ Studied Using Soft X-ray Absorption Spectroscopy and Magnetic Circular Dichroism. Phys. Rev. Lett..

[26] Hu Z (2012). Spin-state order/disorder and metal–insulator transition in GdBaCo_2_O_5.5_: experimental determination of the underlying electronic structure. New J. Phys..

[27] Huang Q (1994). Neutron Powder Diffraction Study of the Crystal Structures of Sr_2_RuO_4_ and Sr_2_IrO_4_ at Room Temperature and at 10 K. J. Solid State Chem..

[28] Mikhailova D (2017). Charge Transfer and Structural Anomaly in Stoichiometric Layered Perovskite Sr_2_Co_0.5_Ir_0.5_O_4_. Eur. J. Inorg. Chem..

[29] Chen J-M (2014). A Complete High-to-Low spin state Transition of Trivalent Cobalt Ion in Octahedral Symmetry in SrCo_0.5_Ru_0.5_O_3-δ_. J. Am. Chem. Soc..

[30] Groot FMF (1994). de X-ray absorption and dichroism of transition metals and their compounds. J. Electron Spectrosc. Relat. Phenom..

[31] Tanaka A, Jo T (1994). Resonant 3*d*, 3*p* and 3*s* Photoemission in Transition Metal Oxides Predicted at 2*p* Threshold. J. Phys. Soc. Jpn..

[32] Udd = 5.5 eV, Upd = 7.0 eV; ∆ = 2.0 eV; The Slater integrals were reduced to 80% of their Hartree-Fock values.

[33] Herrero-Martín J (2012). Spin-state transition in Pr_0.5_Ca_0.5_CoO_3_ analyzed by x-ray absorption and emission spectroscopies. Phys. Rev. B.

[34] Vankó G, Rueff J-P, Mattila A, Németh Z, Shukla A (2006). Temperature- and pressure-induced spin-state transitions in LaCoO_3_. Phys. Rev. B.

[35] Chen JM (2014). Evolution of spin and valence states of (Pr_0.7_Sm_0.3_)_0.7_Ca_0.3_CoO_3_ at high temperature and high pressure. Phys. Rev. B.

[36] Tsutsumi K, Nakamori H, Ichikawa K (1976). X-ray Mn *Kβ* emission spectra of manganese oxides and manganates. Phys. Rev. B.

[37] Oka K (2010). Pressure-Induced Spin-State Transition in BiCoO_3_. J. Am. Chem. Soc..

[38] Kozlenko DP (2007). Temperature- and pressure-driven spin-state transitions in LaCoO_3_. Phys. Rev. B.

[39] Vogt T, Hriljac JA, Hyatt NC, Woodward P (2003). Pressure-induced intermediate-to-low spin state transition in LaCoO_3_. Phys. Rev. B.

[40] Baier J (2005). Spin-state transition and metal-insulator transition in La_1−x_Eu_x_CoO_3_. Phys. Rev. B.

[41] Cwik, M. The Interplay of Lattice, Spin, and Charge Degrees of Freedom in Layered Cobaltates. Ph.D. Dissertation, Universitat of Cologne, Cologne, 2007.

[42] Mikhailova D (2014). Oxygen-driven competition between low-dimensional structures of Sr_3_CoMO_6_ and Sr_3_CoMO_7−δ_ with M = Ru, Ir. Dalton Trans..

[43] Doumerc J-P (2001). Crystal structure of the thallium strontium cobaltite TlSr_2_CoO_5_ and its relationship to the electronic properties. J. Mater. Chem..

[44] Doumerc J-P, Grenier J-C, Hagenmuller P, Pouchard M, Villesuzanne A (1999). Interplay between Local Electronic Configuration and the Occurrence of a Metallic State: An Experimental Approach. J. Solid State Chem..

[45] Kini NS, Strydom AM, Jeevan HS, Geibel C, Ramakrishnan S (2006). Transport and thermal properties of weakly ferromagnetic Sr2IrO4. J. Phys.: Condens. Matter.

