# Supplementary material for: Relation between the Co-O bond lengths and the spin state of Co in layered Cobaltates: a high-pressure study
Source: Sci Rep. 2017 Jun 16;7:3656. doi: 10.1038/s41598-017-03950-z (PMC5473858; doi:10.1038/s41598-017-03950-z)
Supplement: Supplementary file 1 — Supplementary information. [file 41598_2017_3950_MOESM1_ESM.doc]

**Supplementary Information**

**Relation between the Co-O bond lengths and the spin state of Co in layered Cobaltates: a high-pressure study**

**Yi-Ying Chin^1^, Hong-Ji Lin^1^, Zhiwei Hu^2^, Chang-Yang Kuo^2^, Daria Mikhailova^2,3,4^, Jenn-Min Lee^1^, Shu-Chih Haw^1^, Shin-An Chen^1^, Walter Schnelle^2^, Hirofumi Ishii^1^, Nozomu Hiraoka^1^, Yen-Fa Liao^1^, Ku-Ding Tsuei^1^, Arata Tanaka^5^, Liu Hao Tjeng^2^, Chien-Te Chen^1^, and Jin-Ming Chen^1^**

^1^National Synchrotron Radiation Research Center, Hsinchu, 30076, Taiwan

^2^Max Planck Institute for Chemical Physics of Solids, Dresden, D-01187, Germany

^3^Karlsruhe Institute of Technology (KIT), Institute for Applied Materials (IAM), Eggenstein-Leopoldshafen, D-76344, Germany

^4^Institute for Complex Materials, IFW Dresden, Dresden, D-01069, Germany

^5^Department of Quantum Matter, ADSM, Hiroshima University, Higashi-Hiroshima, 739-8530, Japan

Correspondence and requests for materials should be addressed to J.M.C. (email: [jmchen@nsrrc.org.tw](mailto:jmchen@nsrrc.org.tw)) or H.J.L. (email: [hjlin@nsrrc.org.tw](mailto:hjlin@nsrrc.org.tw))

Figure S1. The results of the resistivity measurements on Sr_2_Co_0.5_Ir_0.5_O_4_ as well as those of Sr_2_IrO_4_ for comparison.^45^
